# Supplementary material for: In Vitro and In Vivo Regulation of SRD5A mRNA Expression of Supercritical Carbon Dioxide Extract from Asparagus racemosus Willd. Root as Anti-Sebum and Pore-Minimizing Active Ingredients
Source: Molecules. 2022 Feb 24;27(5):1535. doi: 10.3390/molecules27051535 (PMC8911958; doi:10.3390/molecules27051535)
Supplement: Supplementary file 1 [file molecules-27-01535-s001.zip › molecules-1559025-supplementary.pdf]

Table S1 Precision of the method, linearity data for calibration curves and retention time (RT) of reference phenolic compounds studied.

Compounds	Mass	Linearity (ug/ml)	Retention time (min)	Regression equation	R^2	LOD (ug/ml)	LOQ (ug/ml)
Gallic acid	170.12	12.5-100	5.491	Y=22646x+125831	0.9981	0.37	1.12
Catechin	290.26	12.5-100	7.643	Y=22734x-58991	0.9950	0.20	0.62
Epigallocatechin gallate	458.37	12.5-100	8.274	Y=19278x+55634	0.9999	0.06	0.19
Epicatechin	290.26	12.5-100	9.003	Y=22488x+65129	0.9996	0.71	1.23
Caffeic acid	180.16	12.5-100	10.326	Y=31241x+235517	0.9986	0.05	0.16
Rutin	610.51	12.5-100	14.603	Y=36249x-34782	0.9999	0.09	0.28
p-Coumaric acid	164.04	12.5-100	16.579	Y=13162x+82611	0.9993	0.19	0.57
Naringin	580.54	12.5-100	18.848	Y=32805x+50054	0.9987	0.12	0.36
Rosmarinic acid	360.31	12.5-100	20.158	Y=59331x+331236	0.9986	0.34	1.02
Quercetin	302.23	6.25-50	23.378	Y=63129x+221430	0.9964	0.01	0.04
Naringenin	272.25	12.5-100	25.210	Y=59542x-195443	0.9977	0.05	0.15

LOD: limits of detection; LOQ: limits of quantification
